# Progress in Medicine

**Published:** 1899-07-08

**Authors:** 


### Progress in Medicine.

### DISEASES OF THE DIGESTIVE ORGANS.

**Duodenal Ulceration.**—These ulcers are difficult of diagnosis, and comparatively rare. Burwinkel[1] reports five cases recognised during life. Among the symptoms was profuse melæna without any blood in the vomit, while malignant or tuberculous ulceration and affections of the lower bowel could be excluded. The pain was felt in the situation of the duodenum, came on two or three hours after a meal, and had been preceded for a long while by dyspeptic troubles. The author's cases, like the majority of others, occurred in men of about 40. A fatal termination is more frequent than in gastric ulcer. Thus perforation, with peritonitis or the erosion of a large vessel, is not uncommon, while stenosis of the duodenum has been observed. Two cases were met with recently in the Bristol General Hospital. In one the patient was a man of 62, who had suffered from dyspepsia with loss of flesh for some time. He complained of attacks of pain in the neighbourhood of the gall-bladder, but on examination no abnormality could be discovered in the liver, kidneys, or bowels. One morning profuse melæna came on and ended fatally. The other patient, a male of 50, of intemperate habits, was found by the police lying on the ground vomiting and suffering great pain. He was brought in on a stretcher, vomited a small amount of coffee-ground material at intervals, and spoke of having a similar attack previously. There was pain and tenderness in the hepatic region, and next day the abdomen became distended, and the patient died from peritonitis. A perforated ulcer was found in the first part of the duodenum, as well as evidences of recovery from a previous perforation.

**Dysentery.**—Buchanan[2] gives the results of treatment of 102 cases with magnesium sulphate. He employs a mixture of 15 grains to the drachm with ginger and sulphuric acid. One or two drachms are given every two hours, or half an ounce four times a day. This amount he gives as long as the stools remain yellow and soft, and until the mucus and blood have been absent two days; but if watery discharges appear it is stopped at once. It is not beneficial in chronic cases, which he treats with two or four drachms of castor oil in milk twice a day, magnesia in exacerbations, santonin (grains v.) if entozoa are suspected, and a milk diet. Relapses are frequent if chills or errors of diet are permitted. Dalgetty[3] recommends a mustard blister to the stomach, followed by 20 minims of laudanum. Ten minutes afterwards he orders four pills, freshly made, each containing 10 grains of powdered ipecacuanha, and makes the patient lie still for two hours without any food or drink. This treatment is repeated in eight hours if there is no improvement, or a smaller dose is given afterwards if the patient is better.

**Membranous Enteritis.**—Max Einhorn[4] reviews the literature of this disease, which was ascribed by Rothmann originally to an increased secretion of the glands of the colon following on continued constipation, the secretion consisting of mucus only. Even now Einhorn confesses that the causation is uncertain. Neurotic women are, perhaps, the most numerous patients, and the author found that 12 sufferers out of 20 had marked enteroptosis. Ewald had previously noted some relationship between the two diseases, a cause for which it is not difficult to see. Einhorn now points out that many of the patients also suffer from achylia gastrica, which is a rather rare disease. He notes the attacks every month or so of violent abdominal pains with the passage of mucous masses, and sometimes straining and diarrhœa following after a period of constipation. During the attacks he merely orders rest in bed, with opium, but between them he injects eight to 15 ounces of warm oil into the bowel every night, which the patient has to retain. This is continued for three weeks, and gradually reduced in frequency till one only is given every week for five or six months. A daily action of the bowels is maintained, which the oil assists, and the final results are said to be most satisfactory. J. M. Fladger[5] records a case in a child of five years, following a febrile attack and lasting some three months, which was treated with injections of nitrate of silver. The question arose whether this was a case of intestinal diphtheria of unusual duration, especially as it was complicated with great loss of power in the limbs. In cases of ulcerative colitis Richter[6] advises an astringent enema in the early morning after the contents of the bowels have been removed by irrigation. These enemata may contain bismuth and tanalbin or decoction of bilberries. Three curious outbreaks of epidemic diarrhœa in the wards of St. Bartholomew's Hospital have taken place, as many as 146 persons being attacked in one night. The last of these was traced to a most unexpected source, namely, hot rice pudding. In this and in the stools of some of the patients which were examined was found the bacillus enteritidis sporogenes. This is one of the most resistant organisms known, and will stand a temperature of 100 deg. C., though it is only virulent in exceptional circumstances. If Dr. Andrewes'[7] reasoning is correct, this was an outbreak of a most unusual character. In obstinate cases of chronic colitis colotomy is recommended in order to give complete rest to the bowel. Three cases were brought forward by Hale White and Golding Bird[8] at the Clinical Society. A woman, aged 36, had suffered from membranous colitis for 20 years, and the operation resulted after 18 months in a complete cure. In a woman, aged 31, similar relief was obtained. The third patient, a man, had suffered for eight years from diarrhœa and melæna. A cæcotomy resulted also in a cure. They recommend right-sided colotomy for intractable cases of membranous colitis, for similar cases of ulcerative colitis, and for idiopathic dilation of the colon. In all their cases the passage of membrane ceased as soon as the colon was stitched to the skin and before it was opened, showing that the affection is due to reflex action. An outbreak of diarrhœa and vomiting affecting eight persons, with one fatal result, after a meal of pork, occurred recently in Glasgow. C. M'Clure[9] isolated from the stools the proteus vulgaris, which had been occasionally found before in a few similar instances which he refers to.

**Auto-intoxications.**—These are divided by Sidney Martin[10] into (*a*) those of intestinal origin, (*b*) those seen in chronic diseases, and (*c*) those connected with glands. The first class comprises cases of bacterial decomposition, which are not true auto-intoxications, but ordinary poisoning. There are also cases of the failure of digestion where, perhaps, albumoses are absorbed, instead of the proper proteid into which they should have been changed. In class *b* the poisons, *e.g.*, in diabetes and uræmia, have not been isolated, and indeed no poison has been yet discovered which is formed from proteids without the action of bacteria. Cases arising from the removal of a gland such as the thyroid may be due either to the absence of an internal secretion, or to the fact that some poison is no longer eliminated. Mott believes that the poison in general paralysis is cholin, which is produced by the breaking up of phosphoric fats. He shows that many of the symptoms may be due to its presence. The invasions of the internal organs by bacteria from the intestines is discussed by Birch-Hirschfield.[11] He considers it highly improbable that the bacillus coli can pass through the healthy intestinal wall, but if lesions of the wall occur they effect an entrance, as well as during the death struggle, and again some ten hours or later after death. They are then conveyed along the lymph and blood channels, or through the mucous lining to various organs. Their virulence in the cadaver affords no measure of their virulence during life.

**Idiopathic Dilatation of the Colon.**—Instances are found where the large intestine is greatly distended without any discoverable cause of obstruction. H. Sinigar[12] reports two cases, both of which were in aged women. The first suffered from bronchitis, with great abdominal distention, and gave a history of simple constipation which was successfully treated. After her death from bronchitis the entire colon was found to be enormously dilated, with slight muscular hypertrophy. There was no evidence of the impaction of scybala or of any growth or cause of obstruction. The second patient died from obstruction after obstinate constipation. An operation on the colon failed to relieve her.

The rectum was found distended to a circumference of 26 inches, and occupied the whole of the front of the abdomen. No scybala were present. The colon was of normal size. The writer inclines to the view of Hale White that these cases are analogous to simple dilatation of the stomach, and due to some failure of innervation of the bowel.

**Hæmorrhagic Ascites.**—J. M. Finny[13] records a case where the fluid required repeated tapping, and as much as six and a half gallons were thus removed in three weeks. The colour was that of blood, and the sediment amounted to about a ninth of the whole, and contained blood cells and a few large round ones from the epithelium. The patient was only ill three months, and retained his appetite and activity till shortly before his death. A sarcomatous growth of the omentum was the cause of this condition, which appears to be very rare. Milky ascites may be due to malignant growths, but is also found after injury and where the chyle ducts have been subjected to pressure. Ceconi[14] divides these cases into: (*a*) Chylous, where true chyle is present; (*b*) chyliform, where there is fatty degeneration of cells from malignant disease—no increase in the suspended matter occurs in this case if a diet rich in fats is given, while the opposite is seen in the first group; (*c*) occasionally a fluid derived from both sources may be present; (*d*) lastly, fluids of a similar appearance, but without fat, may be due to certain albuminoid matters derived from the blood serum.

**Appendicitis.**—E. Maylard[15] speaks against indiscriminate operation, and points out the large number of cases which subside without active treatment. In the first 24 hours of a simple acute attack he would advise copious enemata carefully given, the hourly administration of sulphate of magnesia until the bowels act, and linseed poultices, but no opium. Not only milk, but broth and meat jelly are given by the mouth, until by the fourth day definite improvement, or marked local symptoms are seen. Of course, this only applies to cases without complications. Indeed, the suddenness with which complications may occur necessitates the surgeon knowing the symptoms of a case from the outset. He regards no case as perfectly cured in which there is not complete absence of any fulness deep in the iliac fossæ, the absence of all tenderness, and all discomfort whatever in active movement. If, at the end of a month, there are any such indications he advises operative interference while the disease is quiescent. R.I. Morris[16] warns us against diagnosing as appendicitis cases of sub-acute inflammation near the appendix in lithæmia, cases of neuralgia, and cases of colitis. He records as errors in diagnosis a case of adhesions about the cæcum due to former typhoid; one of peritoneal tuberculosis affecting especially the appendicular region; a case of cancer of the cæcum; one of adhesions of the appendix, due to salpingitis; one of pneumonia; another of hysteria; and one of peritonitis following measles. These were the only cases out of 228 operated on for appendicitis where that disease proved absent. Mitchell, of the Johns Hopkins Hospital,[17] finds that in 1,400 recorded cases of appendicitis foreign bodies were present in 7 per cent. of the whole, while fæcal concretions existed in 45 per cent. of those in which a definite record was made. Among foreign bodies pins and shot seem to be the most frequent. Hæmatemesis in obstruction of the bowels is exceedingly rare, but two cases are recorded by A. Tietze.[18] The first case was due to compression by adhesion from an old perityphlitis and intense congestion of the gut, while in the second the intestines were strangulated by the edge of the omentum.

1 Brit. Med. Jour., Feb. 25. 2 Brit. Med. Jour., Feb. 11. 3 Brit. Med. Jour., April 15. 4 Med Rec., Jan. 28. 5 Charlotte Med. Jour., Feb. 6 Brit. Med. Jour., April 5. 7 Lancet, Jan. 7. 8 Lancet, May 20. 9 Glasgow Med. Jour., Dec. 10 Lancet, Mar. 4. 11 Med. Chron., April. 12 Lancet, Mar. 11. 13 Dublin Jour. Med. Sci., Jan. 14 Brit. Med. Jour., Feb. 11. 15 Glasgow Med. Jour., Mar. 16 New York Med. Jour., April 8. 17 Med. Rec., April 1. 18 Med. Rec., Dec. 31.

